# Genotypes and Genomic Regions Associated With *Rhizoctonia solani* Resistance in Common Bean

**DOI:** 10.3389/fpls.2019.00956

**Published:** 2019-07-24

**Authors:** Atena Oladzad, Kimberly Zitnick-Anderson, Shalu Jain, Kristin Simons, Juan M. Osorno, Phillip E. McClean, Julie S. Pasche

**Affiliations:** ^1^Department of Plant Sciences, North Dakota State University, Fargo, ND, United States; ^2^Department of Plant Pathology, North Dakota State University, Fargo, ND, United States

**Keywords:** *Phaseolus vulgaris*, GWAS, quantitative resistance, rhizoctonia, root rot

## Abstract

*Rhizoctonia solani* Kühn (teleomorph *Thanatephorus cucumeris*) is an important root rot pathogen of common bean (*Phaseolus vulgaris* L.). To uncover genetic factors associated with resistance to the pathogen, the Andean (ADP; *n* = 273) and Middle American (MDP; *n* = 279) diversity panels, which represent much of the genetic diversity known in cultivated common bean, were screened in the greenhouse using *R. solani* anastomosis group 2-2. Repeatability of the assay was confirmed by the response of five control genotypes. The phenotypic data for both panels were normally distributed. The resistance responses of ∼10% of the ADP (*n* = 28) and ∼6% of the MDP (*n* = 18) genotypes were similar or higher than that of the resistant control line VAX 3. A genome-wide association study (GWAS) was performed using ∼200k single nucleotide polymorphisms to discover genomic regions associated with resistance in each panel, For GWAS, the raw phenotypic score, and polynomial and binary transformation of the scores, were individually used as the input data. A major QTL peak was observed on Pv02 in the ADP, while a major QTL was observed on Pv01 with the MDP. These regions were associated with clusters of TIR-NB_ARC-LRR (TNL) gene models encoding proteins similar to known disease resistance genes. Other QTL, unique to each panel, were mapped within or adjacent to a gene model or cluster of related genes associated with disease resistance. This is a first case study that provides evidence for major as well as minor genes involved in resistance to *R. solani* in common bean. This information will be useful to integrate more durable root rot resistance in common bean breeding programs and to study the genetic mechanisms associated with root diseases in this important societal legume.

## Introduction

Common bean (*Phaseolus vulgaris* L.) is one of the most important cultivated grain legumes for human consumption in the world (United States Dry Bean Council, 2017). Cultivated common bean evolved from two major wild genepools, Middle American and Andean, which were established in Mexico ∼110 kyr ago (Mamidi et al., 2011; Bitocchi et al., 2013; Schmutz et al., 2014). Differences between the two genepools include plant and seed morphology, mineral content, responses to diseases, and DNA polymorphisms (Gepts et al., 1986; Blair et al., 2006, 2009). The cultivated genotypes of the two genepools are further divided into races based on morphological, agronomical, and molecular differences (Singh et al., 1991). The Middle American genepool consists of four races: Durango, Jalisco, Mesoamerican, and Guatemala (Singh et al., 1991; Beebe et al., 2001; Blair et al., 2009). The Andean genepool is divided into three races including Nueva Granada, Peru, and Chile (Singh et al., 1991). Middle American beans are primarily grown from North America, through Central America, and into northern South America. These include the pinto, navy, great northern, black, and small red beans market classes. Kidney beans, from race Nueva Granada of the Andean gene pool, are also prevalent in the US (United States Dry Bean Council, 2017) and Africa. Collections of 396 and 280 *P. vulgaris* accessions, referred as the Andean Diversity Panel (ADP; Cichy et al., 2015) and Middle American Diversity Panel (MDP; Moghaddam et al., 2016), were created to reflect the genetic diversity in the two major gene pools and facilitate gene pool specific genetic analyses.

The genetic diversity present in both gene pools can be useful to identify potential sources of resistance against the basidiomycete pathogen *Rhizoctonia solani*. *R. solani* is a global soil-borne pathogen that has a broad host range including rice, ginger, corn, sugar beet, cauliflower, pine, common bean, soybean, alfalfa, tomato, potato, onion, snap bean, cotton, and peas (Anderson, 1982; Sneh et al., 1991; Pannecoucque and Hofte, 2009; Bolton et al., 2010). *R. solani* is not a true species but rather a species complex comprised of 15 anastomosis groups (AGs) (Carling et al., 2002; Stodart et al., 2007; Bolton et al., 2010). The 15 AGs are defined by morphology, pathogenicity, virulence, DNA polymorphisms, and their ability to anastomose (Carling et al., 2002; Stodart et al., 2007; Bolton et al., 2010). Hyphal anastomosis, a genetically controlled event between compatible genotypes, involves fusion of hyphal tips from distinct individuals and subsequent successful hyphal growth. The two most frequently isolated AGs from crops grown in the Northern Great Plains region of the US are AG 2-2 and AG 4. AG 2-2 is the most aggressive on common bean (Engelkes and Windels, 1996; Mathew et al., 2012).

*Rhizoctonia solani* severely impacts seed yield of common bean, resulting in upwards to 100% seed yield loss (Hagedorn, 1994; Singh and Schwartz, 2010). Root symptoms caused by *R. solani* include sunken, water-soaked, reddish-brown lesions with a range of sizes resulting in pre-emergence damping off of seedlings (Reddy et al., 1993; Hagedorn, 1994). When the disease is severe, stunting and premature plant death can occur. Although *R. solani* spreads via airborne basidiospores when the teleomorph (sexual) stage is present, this is uncommon in the field (Godoy-Lutz et al., 2003). Vegetative mycelia and sclerotia of the asexual stage colonize the soil environment and can overwinter in the soil for several years (Godoy-Lutz et al., 2003). Soil-borne *R. solani* spreads from plant to plant through the formation of mycelial bridges between plant roots and infested soil debris.

Genetic resistance is considered the most cost effective and sustainable management of root rots in common bean (Tu, 1992; Park and Rupert, 2000; Abawi et al., 2006). However, genetic analysis of resistance to *R. solani* in common bean has been limited (Muyolo et al., 1993; Peña et al., 2013; Conner et al., 2014; Adesemoye et al., 2018). Field studies are challenging because of the presence of more than one soil-borne root pathogen (Conner et al., 2014). Greenhouse studies were carried out to discover resistant genotypes (Muyolo et al., 1993; Peña et al., 2013; Adesemoye et al., 2018) but were not followed up by genetic analysis. While these studies provided valuable information, wide-scale resistance screening across the full range of cultivated genotypes, such as those comprising the ADP and MDP, have not been completed.

Genetic resistance to *R. solani* is quantitative in nature (Zhao et al., 2005). While environmental factors can greatly affect phenotypic responses, it is notoriously difficult to screen for resistance to soil-borne pathogens due to the struggle of maintaining constant moisture and temperatures within a soil environment. Therefore, to obtain consistent and reproducible phenotypic data for a set of test genotypes, the screening protocol for root rot pathogens must generate predictable reactions to the pathogen from a set of control lines expressing variable levels of resistance.

The development of reference genome sequences and single nucleotide polymorphism (SNP) marker technology has rapidly improved our understanding of the genetics and molecular mechanisms associated with resistance to plant pathogens. One approach is to compare the frequency of the genetic variants across the genome in a large number of resistance and susceptible genotypes and identify the alleles correlated with each haplotype (Koboldt et al., 2013). Genome-wide association studies (GWASs) utilize linkage disequilibrium (LD) along with knowledge of the structure of a given population to discover important genetic factors. Large and highly diverse association panels have unique recombination histories enabling the detection of large and small genetic effects associated with response of the host to a pathogen. GWAS analyses are emerging as a method to discover disease resistance loci in common bean (Shi et al., 2011; Hart and Griffiths, 2015; Perseguini et al., 2016; Zuiderveen et al., 2016; Tock et al., 2017). To date, the GWAS studies aimed at discovering loci associated with resistance to root pathogens in common bean has been limited to *F. solani* in the ADP and to a lesser extent *Pythium* spp. (Vasquez-Guzman, 2016; Soltani et al., 2018). The focus here is to dissect the genetic architecture of resistance to *R. solani* in common bean independently in the two major gene pools of common bean using a GWAS approach.

From an analytical perspective, the root rot phenotype is scored quantitatively (Park and Rupert, 2000; Román-Avilés and Kelly, 2005; Conner et al., 2014; Hagerty et al., 2015). However, in breeding programs, after evaluations of the phenotypic scores of the pre-breeding lines, breeders usually classify lines categorically as resistant, moderately resistant, or susceptible. This approach effectively transforms the quantitative phenotype data into a qualitative phenotype. One feature of this study is to determine if quantitative and qualitative phenotypic data detect the same or different genomic regions associated with the disease. Here, we address this question by applying GEMMA (Zhou and Stephens, 2012, 2014) algorithms on the raw phenotypic score, and transformations of the scores into polynomial and binary scores. The advantage of using the GEMMA package is that it performs GWAS for any phenotypic distributions by performing a generalized linear model instead of general linear model. In the generalized linear model, the associated *p*-value for each marker is based on the likelihood changes when a single marker is added to the model and therefore, it is suitable for response variables following different distributions. Since GEMMA considers multiple linked functions to transform the response variable to the appropriate distribution, different relationships between the linear model and the response variable can be tested. Here, we tested different approaches with different data sets to conduct GWAS. The first approach is the standard mixed-model approach based on the raw mean disease scores. The raw data was also transformed into three class polynomial data (resistant, moderately resistant, and susceptible) and two-class binary data (resistant or susceptible) based on the response of the check genotypes in the analysis. The transformation may allow the discovery of major genetic effects. Markers associated with those effects may be mobilized as useful breeding tools.

The objectives of this research were to: (1) demonstrate reproducibility of a greenhouse evaluation system for *Rhizoctonia* root rot in common bean; (2) characterize resistance to *R. solani* within the ADP and MDP; (3) discover genomic regions associated with resistance based on raw and transformed phenotypic data using GWAS; and (4) identify candidate genes underlying resistance to *Rhizoctonia* root rot in common beans.

## Materials and Methods

### Inoculum Preparation

One *R. solani* AG 2-2 isolate collected from common bean grown in the Red River Valley region of North Dakota was utilized for screening common bean germplasm under greenhouse conditions. The isolate was purified via micro-excision of a single hyphal tip and previously confirmed as *R. solani* AG 2-2 via amplification of the rDNA ITS region using ITS1, 5.8S, and ITS2 with primers ITS4 (TCCTCCGCTTATTGATATGC) and ITS5 (GGAAGTAAAAGTCGTAACAAGG) (White et al., 1990; Mathew et al., 2012). The isolate was grown at 20 ± 2°C for 14 days on 0.5× potato dextrose agar (PDA: 19.5 g of potato dextrose agar, 7.5 g of agar, 1 L of distilled H_2_O) amended with streptomycin and neomycin, both at a concentration of 50 mg/L. Large metal trays (30.48 cm × 5.80 cm × 6.35 cm) were filled with 1.5 kg of wheat kernels. Tap water was added to the trays to a depth of approximately 8 cm to completely cover the wheat kernels. The wheat kernels were soaked for approximately 15 h at 22 ± 2°C, water was drained from the trays, trays were covered with aluminum foil and autoclaved at 121°C for 2 h, cooled for 24 h at room temperature, and autoclaved again for 2 h. After the wheat kernels were cooled overnight, they were aseptically inoculated with one cm square sections of 0.5× PDA containing mycelia of *R. solani* from eight (100 mm diameter) Petri plates. Trays were covered with aluminum foil, and the wheat was colonized over a period of 2 weeks at 22 ± 2°C. The grain was aseptically mixed twice a week to ensure uniform colonization. The trays were emptied onto butcher’s paper and spread out to dry on greenhouse benches. The dry, colonized wheat was collected, sieved, and stored in the freezer until use.

### Greenhouse Evaluations

A total of 279 MDP and 273 ADP common bean genotypes were evaluated for resistance to *R. solani* under greenhouse conditions using an augmented randomized complete block design. Each genotype was replicated three times (1 replicate = 1 pot). Each pot contained three seeds of a single genotype. To control for differences in root architecture and color across accessions when evaluating for root rot, one pot containing three seeds and no inoculum was included as a negative control. Four-inch plastic pots with drainage holes were filled to the top rim with Pro-mix LP15 potting soil (Premier Tech Horticulture, Quakertown, PA, United States). One *R. solani* colonized wheat kernel was placed 2 cm below the bean seed, which was planted at a depth of approximately 2 cm. The seeds were covered with soil, and the soil was watered. Plants were watered daily and maintained over a 14 days period in a greenhouse under 16 h of light at 22 to 25°C. To ensure consistent environmental conditions, the ADP was screened in six sub-groups consisting of 45 or 46 lines. The MDP was divided into four sub-groups consisting of 72 or 73 lines. The control lines Montcalm (susceptible), Cabernet (susceptible), Dynasty (moderately susceptible), GTS104 (moderately resistant), and VAX3 (resistant) were chosen based on preliminary trials and previously published data (Vasquez-Guzman, 2016). All five-control lines were screened along with genotypes from each sub-group of the ADP and MDP. Therefore, each control line was screened a total of 10 times whereas each genotype was screened twice.

### Disease Evaluations

Plants were harvested 2 weeks after planting by carefully lifting the roots from the potting medium, and the roots were washed with warm tap water. Roots were evaluated for disease using a previously developed disease rating scale; 1 = no visible disease symptoms, 9 = approximately 75% or more of the hypocotyl/root tissue is affected with advanced stages of rotting along with significant reduction in root system (Van Schoonhoven and Pastor-Corrales, 1987). To confirm that disease was the result of the soil infestation with *R. solani*, roots from the five control lines were surface sterilized in a 0.8% NaOCl solution for 30 s and placed onto 0.5× PDA amended with streptomycin and neomycin, both at a concentration of 50 mg/L. After 24 h, hyphal tips were placed into 0.5× PDA to obtain clean cultures. Sub-cultures were grown for 1 week, and each isolate was morphologically identified (Sneh et al., 1991). Morphological identification was verified by amplifying DNA extracted from cultures, using the internal transcribed spacer region protocol described above (White et al., 1990).

### Phenotyping Data Analyses

Assay reproducibility was evaluated utilizing the data for the control lines across six ADP and four MDP sub-group evaluations. The mean and standard error (SE) were calculated based on the root rot ratings for each control line. The coefficients of variability (SE of the mean/mean) were calculated, and a one-way ANOVA (α = 0.05) was performed within each control line across all six ADP and four MDP sub-group evaluations (Wong and Wilcox, 2000).

Control lines data from the sub-group evaluations within each of the two panels were utilized to determine if the control lines were appropriate to classify the genotypes as resistant, moderately resistant, or susceptible to *R*. *solani*. Significant differences among control lines were determined by generating estimated relative effects, confidence intervals, and *p*-values using data from only the control lines. Relative treatment effects and the corresponding 95% confidence intervals were calculated using the LD_CI macro in SAS (Domhof and Langer, 2002; Shah and Madden, 2004). Relative treatment effects was determined using the mean ranks according to the following equation:

$${\overset{\wedge }{p}}_{i}=\frac{1}{N}\left({\bar{R}}_{i}-\frac{1}{2}\right)$$

where (${\bar{R}}_{i}$) is the mean rank for each bean line among all the observations within the experiment (*N*). Relative effects range from 0 to 1 (Shah and Madden, 2004). Probability values (α = 0.05) were generated using the confidence intervals and the relative effects to statistically separate the five control lines (Altman and Bland, 2011). The MDP and ADP genotypes were classified as resistant, moderately resistant, or susceptible relative to VAX3, GTS104 and Montcalm using *p-*values generated from relative effects and associated confidence intervals.

### Single Nucleotide Polymorphism Data Sets

Individual Middle American (MA) and Andean genepool HapMaps (Oladzad et al., 2019) were used for GWAS mapping. These HapMaps were generated from genotype-by-sequencing (GBS) reads of 469 MA and 325 Andean genotypes. Initially, the libraries for each gene pool were prepared using the two-enzyme (*MseI* and *Taqα1*), low- pass sequencing SNP set protocol described by Schröder et al. (2016). The libraries were then sequenced at HudsonAlpha Institute for Biotechnology^1^ using Illumine HiSeq sequencer. The details of SNP calling procedure along with variant filtering was published in Oladzad et al. (2019).

The MA HapMap contained 205,293 SNPs, and the Andean HapMap consisted of 260,670 SNPs. The relative number of SNPs, based on chromosome size, are uniformly distributed across all chromosomes throughout the genome in both gene pools.

### Genome-Wide Association Studies

Single nucleotide polymorphisms with a minor allele frequency (MAF) ≥ 5% within each panel (∼128k SNPs for MDP; ∼200k SNPs for ADP) were used for GWAS analysis. The analyses were first performed with the raw phenotypic data. The raw genotype scores, based on the 1 to 9 scale, were averaged over all sub-group evaluations and used as the input data for mixed-model analysis The raw data was also transformed in a three-class polynomial data where genotypes with mean scores < 2.5 were classified as resistant, genotypes with mean scores between 2.5 and 3.5 were classified as moderately resistant, and genotypes scored > 3.5 were considered susceptible. In addition, raw data was transformed into a binary classification where genotypes with mean phenotypic scores ≤ 3.0 were considered resistant, and genotype with mean scores > 3.0 were considered susceptible.

Association analysis was performed with GEMMA (Zhou and Stephens, 2012, 2014) using data from three different phenotypic data sets. For each analysis, fixed, random and mixed models were tested. Population structure was estimated by principal component analysis (PCA) using the R Prcomp function (Price et al., 2006), and the number of PCAs that accounted for 25–50% of the variation was included in the model as a fixed effect. Population relatedness was performed using the centered relatedness algorithm within GEMMA. This matrix was considered a random effect in the models. A Wald test was performed to determine if the SNP effect size was significantly different from zero, and the corresponding *p*-values were determined. The empirical distribution of *p*-values was bootstrapped 10,000 times, and from the resulting distribution, SNPs within the lower 0.01 and 0.1% tails were considered significant. The results were followed by a likelihood-ratio-based R2 (R2LR) analysis (Sun et al., 2010), using the genABEL package in R to calculate the amount of variation explained by the significant SNPs in the model.

### Candidate Gene Identification

The physical region within a ±100 kb window centered on the peak SNPs were chosen to select candidate genes. The potential effects of the SNPs on gene functions within this window were obtained from snpEff database previously developed for all SNPs in each gene pool using version 2.0 *P. vulgaris* reference genome annotation data^2^.

## Results

### Assay Reproducibility

**FIGURE 1:**
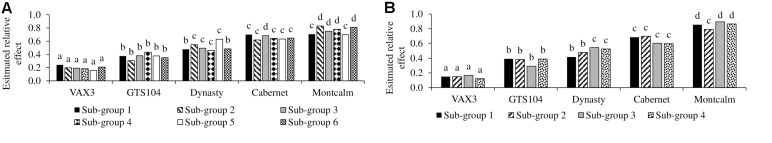
Evaluation of the five control lines against root rot caused by *Rhizoctonia solani* AG 2-2 with sub-group evaluations of *Phaseolus vulgaris* **(A)** Andean Diversity Panel and **(B)** Mesoamerican Diversity Panel. Bars with same letters are not significantly different based on Fischer’s protected least significant difference (α ¡ 0.05).

Genome-wide association study analysis depends on reliable phenotypic data; therefore, the control data was tested for consistency across sub-group evaluations. The phenotypic observations across sub-group evaluations for each control genotype, within diversity panels, were not significantly different (Table 1). In all 10 subgroup evaluations, VAX3 was significantly more resistant to *R. solani* than the other control lines (Figures 1A,B). Montcalm was more susceptible to *R. solani* than the other control lines, except for four comparisons. GTS104 displayed significantly lower root rot severity than Dynasty in six of 10 sub-group evaluations. Dynasty was only significantly different from Cabernet in four of 10 evaluations and was statistically similar to Montcalm in one evaluation.

**TABLE 1:** Mean disease severity (MDS) of five control genotypes evaluated for root rot reaction to *Rhizoctonia solani* AG2- 2 across six sub-group evaluations of the Andean Diversity Panel (ADP) and four sub-group evaluations of the Middle American Diversity Panel (MDP).

**Control genotype**	**Reaction to *Rhizoctonia solani*^a^**	**ADP sub-groups**		**MDP sub-groups**	
		**MDS^b^**	***p*-value^c^**	**MDS^b^**	***p*-value^c^**
VAX3	Resistant	1.9	0.59	1.7	0.44
GTS104	Moderately resistant	2.4	0.15	2.5	0.86
Dynasty	Moderately susceptible	2.8	0.93	2.9	0.43
Cabernet	Susceptible	3.9	0.13	4.1	0.14
Montcalm	Susceptible	4.0	0.17	4.2	0.55

*^*a*^Reacton to *Rhizoctonia solani* based on preliminary trials and previous descriptions (Vasquez-Guzman, 2016).*

*^*b*^Disease severity based on a 1 to 9 scale (Van Schoonhoven and Pastor-Corrales, 1987).*

*^*c*^One-way ANOVA (α < 0.05) comparing MDS across sub-group evaluations for each control genotype within diversity panels.*

### Identification of *Rhizoctonia* Root Rot Resistant Accessions

**FIGURE 2:**
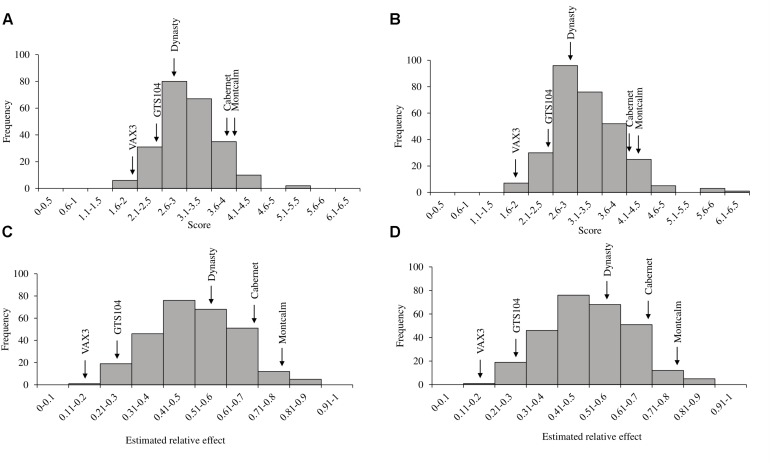
Histograms of phenotypic evaluations of two *Phaseolus vulgaris* diversity panels. Distribution of **(A)** average disease score and **(C)** the estimated relative effects of root rot caused by *R. solani* AG 2-2 in the Andean Diversity Panel (ADP). Distribution of **(B)** average disease score and **(D)** the estimated relative effects of *R. solani* in the Middle American Diversity Panel (MDP). Arrows indicate the average disease score and relative effects of the five control lines used in the study.

*Rhizoctonia* root rot severity data for the ADP were normally distributed and ranged from 2.0 to 5.3 with an overall mean of 3.2 (Figure 2A). Similarly, the average disease severity ranged from 1.9 to 6.2 for the MDP (Figure 2B) and were normally distributed with a mean of 3.1. Corresponding estimated relative effect values, on a scale of 0 to 1 were also normally distributed (Figures 2C,D). Relative effect values ranged from 0.15 to 0.89 and 0.16 to 0.9 for the ADP and MDP, respectively. The estimated relative effects and corresponding confidence intervals of 18 MDP and 28 ADP lines evaluated were not significantly different from the resistant control VAX3, and these lines were categorized as resistant to *R. solani* (Tables 2, 3). Resistant MDP lines belonged to the pinto (*n* = 4) black (*n* = 6), navy (*n* = 4), great northern (*n* = 3), and pink (*n* = 1) market classes. The majority of the resistant ADP lines were purple speckled (*n* = 7) and red mottled (*n* = 4), while the remainder were from various seed coat color/pattern types (Tables 2, 3). Phenotypic values of some lines in the two panels were statistically similar to VAX3 and the moderately resistant control GTS104 (Supplementary Tables S1, S2). All lines statistically similar to the susceptible control Montcalm were significantly different from VAX3. Phenotypic data from two lines were similar to both GTS104 and Montcalm.

**TABLE 2:** Summary of phenotypic data for common bean genotypes within the Andean Diversity Panel (ADP) statistically similar to the resistant control VAX3. Overlapping confidence intervals and *p-*values (α < 0.05)^a^ were used to determine statistical similarity between bean lines and the resistant control (VAX3).

**Line^b^**	**Marketclass/seed color^c^**	**Genotype**	**Mean rank**	**Est. relative effect^d^**	**Confidence interval (95%) for relative effect^e^**	
					**Lower limit**	**Upper limit**
VAX3	Resistant control	VAX3	39.0	0.15	0.08	0.21
ADP1	Red Mottled	ROZI_KOKO	57.8	0.21	0.09	0.42
ADP101	White	Witrood	70.5	0.27	0.15	0.43
ADP124	Red	Maini	86.0	0.32	0.14	0.56
ADP15	Dark Red Kidney	W6_16495	55.8	0.21	0.13	0.31
ADP23	Red	MSHORONYLONI	86.0	0.31	0.14	0.54
ADP390	Dark Red Kidney	PI307808	83.3	0.30	0.13	0.57
ADP428	Pink Cranberry	ColoradodelPais	62.5	0.22	0.07	0.50
ADP430	Pink Mottled	PR1013_3	75.3	0.27	0.15	0.43
ADP435	Red Mottled	RM_05_07	81.9	0.31	0.14	0.54
ADP436	Red Mottled	JB178	81.8	0.31	0.10	0.64
ADP441	Yellow	91_1	55.4	0.20	0.08	0.42
ADP444	Red Mottled	HondoValle25	83.8	0.31	0.14	0.57
ADP460	Purple Mottled	PI331356B	81.1	0.29	0.13	0.52
ADP464	Purple	PI353534B	57.9	0.22	0.15	0.30
ADP468	Manteca	PI527538	74.2	0.27	0.15	0.43
ADP508	Manteca	Calembe	59.2	0.22	0.15	0.30
ADP52	Purple Speckled	RH9	67.9	0.25	0.16	0.37
ADP55	Red Speckled	KABUKU	93.0	0.34	0.17	0.57
ADP56	Purple Speckled	SOYA	97.1	0.38	0.17	0.65
ADP63	Purple Speckled	Soya	86.3	0.33	0.14	0.59
ADP644	Light Red Kidney	FoxFire	90.8	0.34	0.15	0.59
ADP67	Yellow	NJANO	90.7	0.32	0.14	0.59
ADP68	Purple Mottled	Soya	75.8	0.28	0.14	0.48
ADP69	Purple Speckled	SOYA	80.8	0.29	0.13	0.52
ADP74	Purple Speckled	KABLANKETI	96.1	0.36	0.12	0.71
ADP75	Brown	MABUKU	95.4	0.36	0.14	0.66
ADP84	Purple Speckled	KABLANKETI_NDEFU	81.7	0.30	0.10	0.62
ADP86	Purple Speckled	NYAMHONGA_MWEKUNDU	57.5	0.22	0.15	0.30

*^*a*^*p*-Values (α = 0.05) were generated as previously described (Altman and Bland, 2011).*

*^*b*^Identification number for the ADP as previously described (Cichy et al., 2015).*

*^*c*^VAX3 was utilized as the resistant control.*

*^*d*^Estimated relative effect was determined using the mean ranks for each bean line among all the observations within the experiment. Relative effects range between the values of 0 and 1.0 (Shah and Madden, 2004).*

*^*e*^95% confidence intervals were generated from the estimated relative effect values.*

**TABLE 3:** Summary of phenotypic data for bean lines within the Middle American Diversity Panel (MDP) statistically similar to the resistant control VAX3. Overlapping confidence intervals and *p-*values (α < 0.05)^a^ were used to determine statistical similarity between bean lines and the resistant control (VAX3).

**Line^b^**	**Marketclass/seed color^c^**	**Genotype**	**Mean rank**	**Est. relative effect^d^**	**Confidence interval (95%) for relative effect^e^**	
					**Lower limit**	**Upper limit**
VAX3	Resistant control	VAX3	33.9	0.16	0.08	0.16
MDP10	Black	AC_Black_Diamond	64.3	0.24	0.11	0.44
MDP126	Black	Loreto	59.7	0.25	0.11	0.47
MDP350	Black	AC_Harblack	89.8	0.31	0.15	0.53
MDP84	Black	Phantom	98.6	0.36	0.12	0.70
MDP90	Black	B05055	86.7	0.33	0.15	0.59
MDP5	Great Northern	BelMiNeb_RMR_7	63.1	0.24	0.11	0.44
MDP358	Great Northern	Orion	57.6	0.23	0.08	0.52
MDP80	Great Northern	Matterhorn	92.1	0.33	0.15	0.58
MDP339	Navy	Nautica	94.0	0.35	0.14	0.63
MDP55	Navy	Sanilac	74.5	0.28	0.11	0.55
MDP56	Navy	Seafarer	82.5	0.30	0.10	0.64
MDP67	Navy	Laker	86.8	0.33	0.15	0.59
MDP268	Pink	USWA_61	84.8	0.32	0.15	0.56
MDP114	Pinto	Agassiz	70.8	0.26	0.07	0.61
MDP123	Pinto	Sonora	66.9	0.25	0.13	0.43
MDP206	Pinto	NE2_09_8	58.0	0.21	0.08	0.43
MDP22	Black	Shiny_Crow	74.9	0.26	0.09	0.55
MDP79	Pinto	Kodiak	94.3	0.36	0.14	0.67

*^*a*^*p*-Values (α = 0.05) were generated as previously described (Altman and Bland, 2011).*

*^*b*^Identification number for the ADP as previously described (Cichy et al., 2015).*

*^*c*^VAX3 was utilized as the resistant control.*

*^*d*^Estimated relative effect was determined using the mean ranks for each bean line among all the observations within the experiment. Relative effects range between the values of 0 and 1.0 (Shah and Madden, 2004).*

*^*e*^95% confidence intervals were generated from the estimated relative effect values.*

### Association Mapping in Andean Diversity Panel

**FIGURE 3:**
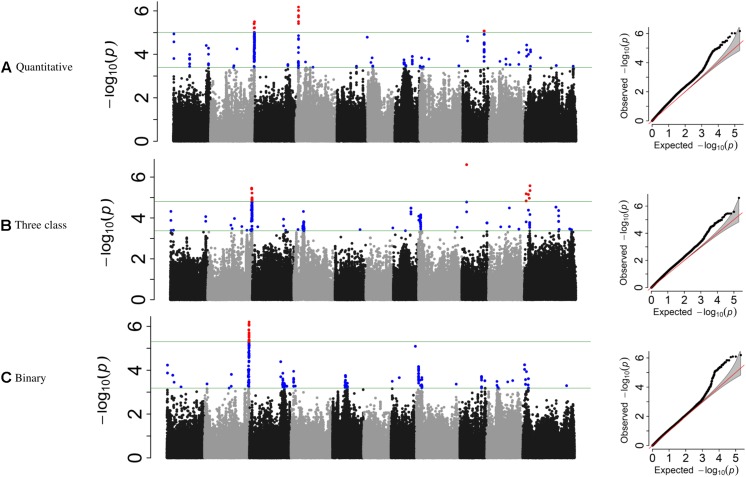
Manhattan plots and corresponding QQ-plots from GWAS analysis of **(A)** quantitative, **(B)** three-class, and **(C)** binary scoring systems in the ADP.

Genome-wide association study analyses were performed with the raw ADP phenotypic data. Across the raw, polynomial (three classes), and binary data set analyses, eight significant intervals (−log_10_(p) ≥ 4.41) containing 128 SNPs were detected (Figure 3 and Table 4). The cumulative variation explained by the three analyses was 22.05, 21.01, and 12.53%, respectively. Fourteen significant SNPs were shared among the three analyses (Table 6). However, several significant SNPs or regions were only detected in one of the analyses (Figure 3). Pv02:48.8–49.41 Mbp was identified as a significant region for all three scoring system analyses. This region contains three gene models, Phvul.002G323704, Phvul.002G323708 and Phvul.002G323712, annotated as TIR-NB_ARC-LRR (TNL) resistance genes (Supplementary Table S3).

**TABLE 4:** GEMMA GWAS results of ADP genotypes for the three phenotypic scoring systems.

**Phenotypic data**	**Type of phenotype**	**Interval**		**Peak SNP**			**% Cumulative variation**
		**Chrom**	**Genomic interval (Mb)**	**Position (Mb)**	*−***Log_10_(p)**	**% Variation**	
Quantitative	Continuous	2	48.88–49.41	S02_49202443	5.49	12.49	22.05
		4	3.88–3.90	S04_3902377	6.17	14.43	
		9	31.61	S09_31614086	5.07	11.32	
Three class	Polynomial	2	48.9–49.41	S02_48896103	5.45	13.12	21.01
		9	5.57	S09_5571299	6.60	14.00	
		11	2.00	S11_2004103	5.18	12.35	
		11	5.06–7.84	S11_5069112	5.57	12.21	
Binary	Binomial	2	48.38–49.41	S02_49236874	6.19	12.53	12.53

Additional SNPs, specific to each phenotypic data set analysis, were also investigated. For the three-class polynomial data GWAS analysis, a gene cluster of disease resistance-responsive, dirigent-like proteins were identified within the interval Pv11:5.06–7.84 Mbp interval. SNP peak at Pv11:6,439,304 bp (*−*log_10_(p) = 4.96) was located 35 kb upstream of this gene cluster. In addition, an ortholog of a putative pathogenesis-related thaumatin protein was detected in this interval at position 5.85 Mbp (Supplementary Table S3).

Genome-wide association study analysis of ADP lines using the raw scoring data detected significant peaks on chromosomes Pv02, Pv04, and Pv09. In this analysis, a novel peak on Pv04 was observed and one SNP (Pv04: 3,902,377 bp) was located within gene model Phvul.004G032900 which is an ortholog of the chorismate mutase gene (*ATCM1*, *CM1*) of Arabidopsis. This peak SNP alone accounted for 14.43% of the phenotypic variation. Another significant SNP at Pv09:31,614,086 bp explained 11.32% of the phenotypic variation mapped 17 kb downstream of a receptor-like kinase gene.

### Association Mapping in Middle American Diversity Panel

**FIGURE 4:**
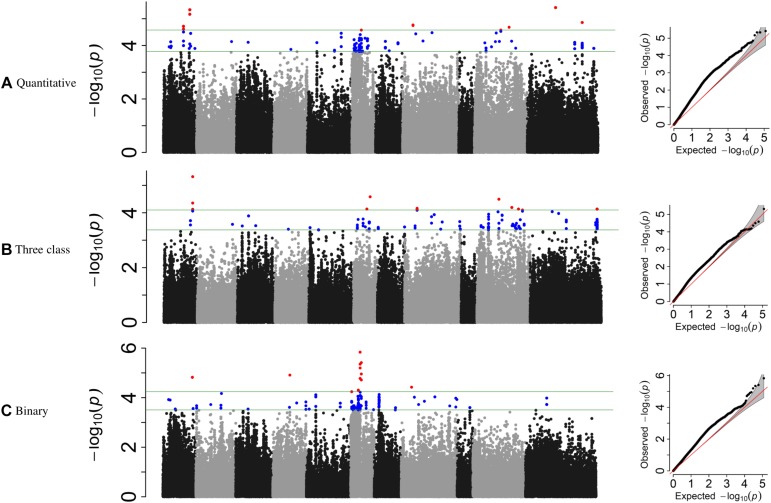
Manhattan plots and corresponding QQ-plots from GWAS analysis of **(A)** quantitative, **(B)** three-class, and **(C)** binary scoring systems in MDP.

For the MDP, 21 significant intervals (−log _10_ (p) ≥ 4.12) containing 38 SNPs were detected for the three data types (Table 5). Several significant regions or single SNP were only detected in one or the other data types (Figure 4). Genomic regions identified using the raw, three-class and binary data sets explained 39.4, 31.7, and 24.6% of the phenotypic variation, respectively (Table 5). Three SNP peaks were shared among different data sets (Table 6).

**TABLE 5:** GEMMA GWAS results of MDP genotypes for the three phenotypic scoring systems.

**Phenotypic data**	**Type of phenotype**	**Interval**		**Peak SNP**			**% Cumulative variation**
		**Chrom**	**Genomic interval (Mb)**	**Position (Mb)**	*−***Log_10_(p)**	**% Variation**	
Quantitative	Continuous	1	23.92	S01_23915969	4.71	7.40	39.40
		1	33.03	S01_33035464	5.34	9.21	
		8	15.26	S08_15258462	4.76	7.90	
		10	19.51	S10_19512563	4.57	7.60	
		10	25.44	S10_25442537	4.68	6.10	
		11	26.41	S11_26407018	5.08	8.90	
		11	41.36	S11_41362555	4.29	7.40	
Three class	Polynomial	1	37.20	S01_37201564	5.31	9.60	31.70
		6	12.20	S06_12029540	4.13	6.10	
		6	17.85	S06_17855757	4.58	6.78	
		8	17.72	S08_36490204	4.16	5.00	
		10	16.40	S10_16404473	4.49	7.60	
		10	25.44	S10_25442537	4.19	6.60	
		10	29.88	S10_29882675	4.13	6.50	
		11	50.59	S11_50585184	4.13	7.40	
Binary	Binomial	1	40.20	S01_40204001	4.82	8.30	24.60
		6	0.57	S06_565179	4.91	6.50	
		6	5.75	S06_5756732	4.30	6.10	
		6	6.89	S06_6897745	5.35	8.00	
		6	8.16	S06_8164695	5.40	7.90	
		8	15.26	S08_15258462	4.42	6.70	

**TABLE 6:** Shared significant SNPs from GWAS results for three scoring system in MDP and ADP gene pool.

**Gene pool**	**SNP**	**Phenotypic data**		
		**Quantitative**	**Binary**	**Three class**
ADP	S02_48896040	✓	✓	✓
	S02_48896103	✓	✓	✓
	S02_48896147	✓	✓	✓
	S02_49202443	✓	✓	✓
	S02_49202459	✓	✓	✓
	S02_49202465	✓	✓	✓
	S02_49229600	✓	✓	✓
	S02_48919593	✓		✓
	S02_49012355		✓	✓
	S02_49025925		✓	✓
	S02_49202378		✓	✓
	S02_49202380		✓	✓
	S02_49224893		✓	✓
	S02_49227910	✓	✓	
MDP	S06_6897745	✓	✓	
	S08_15258462	✓	✓	
	S10_25442537	✓		✓

For the GWAS analyses using raw data, the SNP detected at Pv01: 33,035,464 was adjacent to gene model Phvul.001G109780, an ortholog of 2-isopropylmalate synthase 1 gene. The most significant SNP at position Pv01: 33,035,427 bp explained 9.21% of the phenotypic variation. The SNP peak at Pv08: 15,258,462 shared between the raw and binary data sets is ∼100 kb downstream of a cluster of putative defensin-like (DEFL) family proteins (Supplementary Table S4).

For the three-class polynomial data set, one major gene cluster was detected in close association with significant SNPs at Pv01. A SNP block at Pv01:37.1–37.30 Mbp contained three gene models associated with TNL disease resistance proteins, and a fourth gene model that encodes a leucine-rich repeat protein kinase. The peak SNP identified in this region, is located between two gene models that code for putative disease resistance proteins. Another peak SNP at Pv11: 50,585,184 bp was also in association with a large cluster of at least 11 major NB-ARC domain-containing disease resistance proteins (Supplementary Table S4).

## Discussion

Effective screening for quantitatively inherited traits is challenging, and these challenges are pronounced with root disease traits. Soil-borne pathogens are affected by the environment, and controlling the soil environment in the field during screening of genotypes can be complicated. In addition to soil moisture and temperature, competition from other soil-borne organisms plays an important role in the development of root-rotting pathogens, and these factors are not well-understood. As a result, root-rot incidence and severity are often variable. To our knowledge this is the first case where a statistical confirmation of the control line data ensured that the phenotypic response data for a large collection of genotypes to *R. solani* infection was reliable. While mean maximum disease severity was moderate, statistical validation across control lines provides confidence in both the precision and accuracy of the results presented here. The use of a single, standardized set of control genotypes provides a measure of reproducibility within a single study, but can also allow comparisons among studies across research groups or time.

Prior screenings for resistance to root rot pathogens were performed in the field or greenhouse where the soil was infested with a mixture of fungal mycelium (Román-Avilés and Kelly, 2005; Nicoli et al., 2012; Peña et al., 2013; Conner et al., 2014; Hagerty et al., 2015; Nakedde et al., 2016; Vasquez-Guzman, 2016). In these cases, obtaining a uniform pathogen distribution in the soils was difficult and resulted in variable results. Using a single infested wheat kernel in close proximity to each seed helps to ensure that the roots will interact with the pathogen. The combination of the single infested kernel inoculation method, and a standardized set of control lines, yielded consistent results in screening for genotypes resistant to *R. solani*.

Data generated in these studies supports the use of at least two, and ideally three, control genotypes. Here, VAX3 is statistically validated as the resistant control and is recommended for use in all *R. solani* (AG2-2) root rot evaluations. Recently, a study that screened the ADP under field conditions for root rot resistance also concluded that VAX3 was appropriate as a resistant control line (Vasquez-Guzman, 2016). VAX3 also has been rated as resistant to *Fusarium* root rot among 11 bean lines evaluated (Bilgi et al., 2008). The selection of an effective resistant control line is particularly important because evaluations for disease reaction are frequently focused on identifying or verifying resistance in a genotype. The response of the other four control lines were relatively consistent, but not as clear as VAX3. For eight of the 10 sub-group evaluations performed here, Montcalm was statistically the most susceptible genotype to *R*. *solani*. On that basis, it serves as the most effective susceptible control evaluated. GTS104 was always statistically more susceptible to *R. solani* than VAX3 and nearly always more resistant than both Cabernet and Dynasty.

*Rhizoctonia* root rot has the potential to cause severe economic losses in common bean production regions worldwide. Thus, identifying resistant lines, in several market classes and seed types, and genomic regions conferring resistance to *R. solani* is important to effectively deploy durable genetic resistance. Only a limited number of common bean genotypes have been screened for their response to *R. solani* isolates (Burke and Kraft, 1974; Muyolo et al., 1993; Peña et al., 2013; Conner et al., 2014; Adesemoye et al., 2018). Sources of partial resistance to *R*. *solani* were identified in 275 dry bean lines consisting of commonly grown cultivars, breeding lines, germplasm accessions, and tepary beans in a greenhouse study (Peña et al., 2013). One navy, two black and two cranberry cultivars were partially resistant among 37 common bean cultivars evaluated against root rot pathogens, including *R. solani*, under field conditions (Conner et al., 2014). No resistance to *R. solani* was observed among seven commonly grown common bean cultivars from six market classes screened in a greenhouse study (Adesemoye et al., 2018). The current study is by far the most comprehensive evaluation (*n* = 552 genotypes) of *R*. *solani* resistance in common bean to date. This is a first report of greenhouse screening of the ADP and MDP panels for resistance to *R. solani*. Twenty-eight ADP and 18 MDP genotypes were identified as resistant and will be useful parents for resistance breeding and genetic analysis of the response to the pathogen. Detection of more resistant genotypes in ADP than MDP was surprising as large seeded Andean genotypes generally are considered more susceptible to root rot pathogens (Román-Avilés and Kelly, 2005; Nicoli et al., 2012; Cichy et al., 2015). However, *R*. *solani* has been shown to cause severe damage to navy and pinto beans (Engelkes and Windels, 1996). The new sources of resistance identified in this study provide an avenue to accelerate the introgression of resistance into commercial cultivars. This is particularly true when transferring resistance to *R. solani* between commercial cultivars within the same market class. Commercial cultivars from light red kidney (Foxfire), navy (Nautica, Sanilac, Seafarer, Laker), black (AC Black Diamond, Loreto, AC Harblack, Phantom, Shiny Crow) and pinto (Agassiz, Sonora, Kodiak) market classes exhibited a resistance response similar to VAX3.

Genetic factors associated with the response of common bean to *R. solani* were discovered in two association panels representing the Middle American and the Andean gene pools. The experiment considered the two panels independently because recent reports are demonstrating that different genetic factors or alleles associated with the same phenotype in each gene pool (Schmutz et al., 2014; Soltani et al., 2017, 2018; McClean et al., 2018). The use of panels with a large number of genotypes each analyzed with > 120k SNPs provided the opportunity to discover significant genotype–phenotype association that account for much of the phenotypic variation.

The development of root rot symptoms is controlled quantitatively and is typically scored based on a disease scale (Park and Rupert, 2000; Román-Avilés and Kelly, 2005; Conner et al., 2014; Hagerty et al., 2015). Zhao et al. (2005) also reported that reaction specifically to *R. solani* is controlled by both major and minor genes with additive effects. However, breeders want to discriminate lines as resistant, moderately resistant, or susceptible to the disease. In the current study, we considered this perspective by comparing three different phenotyping data types based on data collected on a 1 to 9 scale. Each data type had a different distribution: continuous, binomial, or polynomial. From the perspective of GWAS analysis, GEMMA algorithms implement an efficient generalized mixed linear model analysis that performs a quantile transformation of any phenotypic distribution by dividing the frequency distribution into equal groups. This protects against model misspecification (Zhou and Stephens, 2012, 2014). GEMMA also offers an improved *p*-value computation (Wald test) that not only reduces the GWAS computational time, but also provides greater power to detect significant SNP associations. Basically, the Wald test is a likelihood estimator of the variances that consequently avoids too many repeated operations for each SNP (Zhou and Stephens, 2014).

In general, several significant associations were observed for both diversity panels. Compared to the MDP, the ADP exhibited fewer significant intervals, with lower *p-*values. The ADP also contained more resistant genotypes. These observations suggest the genetic effects are larger among ADP genotypes than MDP genotypes.

Implementing three different phenotypic scoring systems for the GWAS analysis provided complementary results. The polynomial, three class system detected the most significant SNPs, but the SNPs detected from raw data from the 1 to 9 scale accounted for more phenotypic variation (ADP = 22.05%; MDP = 39.4%). The polynomial system provided a better biological context regarding the candidate genes associated with the resistance. Therefore, the genes discovered with this scoring system may point to major genes associated with the biology of resistance to *R. solani*. The binary data analysis may suffer from missing potentially important genes associated with moderate resistance or missing genes of smaller effect that might be revealed using the full 1 to 9 scoring data.

The GWAS analyses for the three phenotypic approaches identified SNPs within or adjacent to four disease resistance gene clusters (1) TNL disease resistance proteins on the distal end of chromosome Pv02 (ADP) and distal of chromosome Pv01 (MDP); (2) disease resistance-responsive (dirigent-like protein) family on the proximal end of chromosome Pv11 (ADP); (3) putative defensin-like (DEFL) family proteins at position 15.26 of chromosome Pv11 (MDP); and (4) NB-ARC domain-containing disease resistance protein on distal of chromosome Pv11 (MDP).

For the ADP analysis, a common peak on Pv02 was observed for all three data sets. This peak maps near a cluster of gene models that encode TNL disease resistance proteins. In general, TNL proteins directly or indirectly detect pathogen molecules (DeYoung and Innes, 2006). Once the pathogen effector interacts with NLR complex, the LRR domains undergo a conformational change that helps to release ATP from the NB-ARC domain. When ATP is released, TIR domains activate a downstream signal by an unknown mechanism that to the pathogen (DeYoung and Innes, 2006; McHale et al., 2006; Marone et al., 2013). A major peak on chromosome Pv01 (Pv01: 37.1–37.30 Mbp) was observed for the MDP using three-class polynomial data. The peak SNP (37,201,564) for this region was located between two gene models that also putatively encode TNL disease resistance proteins. Another GWAS study in common bean discovered that gene model Phv0l.001G134500 in the same Pv01 cluster was associated with increased resistance to the anthracnose pathogen (Wu et al., 2017). NLR encoding genes are frequently clustered throughout common bean and other plant genomes, a possible result of segmental or tandem duplications (Meyers et al., 2003; Leister, 2004; Monosi et al., 2004). Interestingly, *Rhizoctonia* resistance peaks on Pv01 and Pv02 in both gene pools map within 50 kb of TNL gene model clusters. Kang et al. (2012) discovered that 63% of soybean disease resistance QTL mapped in the regions flanked by NLR encoding genes. Most NLR genes in that study clustered on chromosome Gm06 where four genes were associated with fungal disease resistance. Moreover, a comparative genomic study found that this Gm06 NLR gene cluster was associated with several diseases in soybean and the orthologous common bean cluster on Pv04 was associated with bacterial blight resistance (Ashfield et al., 2012). These studies support our findings that indicate that the TNL gene models in the NLR clusters on Pv02 (for ADP) and Pv01 (for MDP) are reasonable candidates for resistance to *Rhizoctonia* root rot causing pathogens.

A second putative ADP gene cluster located within a significant peak for *R. solani* resistance mapped within a proximal interval on Pv11 using the polynomial data. This cluster is annotated as disease resistance-responsive (dirigent-like protein) family. Zhu et al. (2007) showed that dirigent-like genes in cotton have signaling sequences that encode a class of cell-surface proteins, and these proteins activate receptor-mediated plant defense/immunity mechanism against Verticillium wilt infection. In this interval on Pv11, an ortholog of the putative pathogenesis-related thaumatin superfamily protein was detected. These proteins play a critical role in the plant defense/immunity system. Guerrero-González et al. (2011), discovered that pathogenesis-related genes were differentially expressed in common bean against *R. solani*. Jiang et al. (2015) isolated a pathogenesis-related thaumatin gene from a soybean genotype highly resistant to fungal pathogen *Phytophthora sojae.* Several studies reported that overexpression of thaumatin genes had a positive effect on pathogen tolerance in plants (Liu et al., 1994; Jung et al., 2005; Chassot et al., 2007). Interestingly, the overexpression of this gene enhanced *R. solani* resistance in rice (Datta et al., 1999). The mechanism still is not clear, but it was suggested that this gene might mediate a nuclear transfactor 2 (NTF2) against plant disease (Jiang et al., 2015).

Members of the defensin-like (DEFL) protein family protein were another disease resistance gene cluster found to be associated resistance to *R. solani* in this study. This cluster on chromosome Pv08 was identified in GWAS analysis of MDP using both raw and binary data. DEFL are antimicrobial\cysteine-rich proteins (Giacomelli et al., 2012), and their role in the plant defense response and disease resistance has been reported in several studies (Terras et al., 1995; Gao et al., 2000; Zimmerli et al., 2004). Like NLR genes, DEFL proteins are also located as tandem or segmental duplications (Takeuchi and Higashiyama, 2012). Although the exact mechanism of DEFL is unknown, it has been reported that they are involved in plant innate immunity and pollen tube attractant (Takeuchi and Higashiyama, 2012). Therefore, variation within and among DEFL proteins may affect selection pressure on both reproduction and disease resistance in plants (Swanson and Vacquier, 2002).

The GWAS analysis of the MDP polynomial data also revealed a peak that maps in the NLR encoding gene Phvul.011G192400. The NB-ARC domain contains a functional ATPase region that regulates the activity of the protein-mediated plant resistance. The ARC domain contains a carboxy terminus that can stabilize the domain complex. The ARC domain interacts with the nucleotide-binding domain in order to exchange the nucleotides that are associated with activating ATPase. This may destabilize the complex in a way that it goes under conformational change which in turn reshapes a NB-ARC ATPase and alters resistance specificity (Van Ooijen et al., 2008). Therefore, SNP marker Pv11: 50,585,184 bp, which is inside this gene model might be involved in the nucleotide exchange phase and affect the NB-ARC domain function as a molecular switch.

## Conclusion

Developing resistant varieties is always a primary goal for plant breeders and multiple genotypes were identified with high levels of resistance to *R. solani* in both common bean gene pools. The relevance of these results are validated by the development of assay that demonstrates high reproducibility among control lines. In addition, this is the first GWAS study to uncover the genomic regions associated with *R. solani* resistance in common bean using quantitative and qualitative phenotypic data. The qualitative three class polynomial data yielded the most significant SNPs, however, the raw quantitative data explained more phenotypic variation for both genepools. This study reports strong candidate genes in close proximity to significant SNPs to uncover genetic mechanism of resistance. The significant SNPs within each gene pool can be converted to breeder-friendly markers as efficient and low cost selection tools to identify genotypes resistant to *R. solani*, the causal agent for *Rhizoctonia* root rot.

## Data Availability

All datasets generated for this study are included in the manuscript and/or the Supplementary Files.

## Author Contributions

JP, JO, and KZ-A designed and conceived the experiments. KZ-A and KS performed the experiments in the greenhouse. AO, KZ-A, and SJ performed the data analysis and drafted the manuscript. PM and JP supervised the data analysis. JP, KZ-A, SJ, and AO discussed the results and interpretation of the final data. PM, JP, and JO provided suggestions to improve it. All authors read and approved the final manuscript.

## Conflict of Interest Statement

The authors declare that the research was conducted in the absence of any commercial or financial relationships that could be construed as a potential conflict of interest.

## Footnotes

1 https://hudsonalpha.org/gsc/

2 https://phytozome.jgi.doe.gov/pz/portal.html#!info?alias=Org_Pvulgaris
